# The relationship between timing of onset of menarche and depressive symptoms from adolescence to adulthood

**DOI:** 10.1017/S2045796023000707

**Published:** 2023-09-28

**Authors:** C. Prince, C. Joinson, A. S. F. Kwong, A. Fraser, J. Heron

**Affiliations:** 1 MRC Integrative Epidemiology Unit at the University of Bristol, Bristol, UK; 2 Department of Population Health Sciences, Bristol Medical School, University of Bristol, Bristol, UK; 3 Division of Psychiatry, Centre for Clinical Brain Sciences, University of Edinburgh, Edinburgh, UK

**Keywords:** adolescence, ALSPAC, epidemiology, depression, women

## Abstract

**Aims:**

Girls who experience an earlier onset of menarche than their peers are at increased risk of depressive symptoms in mid-adolescence, but it is unclear if this association persists into adulthood. This study examines whether longitudinal patterns of depressive symptoms from adolescence to adulthood vary according to timing of menarche.

**Methods:**

About 4,864 female participants in the UK Avon Longitudinal Study of Parents and Children provided data on age at onset of menarche (assessed in repeated questionnaires from 8 to 17 years) and depressive symptoms across nine time points (13 to 26 years) using the Short Mood and Feelings Questionnaire. We compared patterns of depressive symptoms in girls with ‘early’ (<11.5 years), ‘normative’ (11.5 to 13.5 years) and ‘late’ (≥13.5 years) menarche using a linear spline multilevel growth curve model adjusted for indicators of socioeconomic position, father absence and body mass index.

**Results:**

Early, compared with normative, menarche was associated with higher levels of depressive symptoms at age 14 (imputed adjusted estimated difference = 0.94, 95% confidence interval [CI] = 0.44, 1.45), but the association attenuated at 24 years (0.24 [−0.72, 1.19]). Late menarche, compared with normative, was associated with a lower level of depressive symptoms at age 14 (−0.69 [−1.10, −0.29]), but this association also attenuated at 24 years (−0.15 [−0.92, 0.62]).

**Conclusions:**

This study did not find a persistent effect of early menarche, compared to normative, on depressive symptoms. However, our findings are consistent with the level of depressive symptoms increasing at the onset of menarche irrespective of timing. The late onset girls ‘catch up’ with their peers who experience menarche earlier in terms of depressive symptoms. Future studies should continue to assess the impact of timing of menarche further into adulthood.

## Introduction

Depression is a leading contributor to the burden of mental health problems in young people and is associated with poor academic performance, substance abuse, eating disorders, dysfunctional interpersonal relationships and suicidal behaviours, and depression in adolescence is also linked to increased depression risk in adulthood (Thapar *et al.*, 2012). Depressive symptoms increase across adolescence, and girls experience a steeper rise in depressive symptoms during early adolescence than boys, and by mid-teens, their rate of depressive symptoms is double that seen in boys (Hankin *et al.*, 1998). A meta-analysis found no further divergence in depression rates between the sexes after adolescence; differences declined and remained stable into adulthood, indicating that adolescence is a key period for the emergence of the unequal sex ratio of depression (Salk *et al.*, 2017). The profound changes of puberty may contribute to the rise in depressive symptoms in girls during adolescence; these changes include biological: fluctuating hormone levels, and the psychosocial consequences of physical development (Angold and Costello, 2006; Ge *et al.*, 2001; Swerdloff and Odell, 1975). There is considerable variation in the age at onset of puberty (Marceau *et al.*, 2011), and girls who experience an earlier puberty than their peers are at greater risk of various adverse psychosocial outcomes, including greater depressive symptoms (Mendle *et al.*, 2007; Skoog *et al.*, 2016). Consistent with the *early timing hypothesis* (Caspi and Moffitt, 1991), studies report a robust relationship between an earlier onset of puberty and an increased risk of depression and depressive symptoms in girls during adolescence (Alcala-Herrera and Marvan, 2014; Galvao *et al.*, 2014; Hamilton *et al.*, 2014; Mendle *et al.*, 2019; Sequeira *et al.*, 2017; Skoog *et al.*, 2016; Wang *et al.*, 2016), and there is evidence that this relationship is causal (Sequeira *et al.*, 2017). It is unclear whether the increased risk of depressive symptoms in early, compared with later, maturing girls persists beyond adolescence. Girls who experience later puberty may eventually ‘catch up’ with earlier maturing girls in late adolescence or early adulthood and eventually display similar levels of depressive symptoms (Stattin and Magnusson, 1990). The *selective persistence hypothesis* predicts that *early* puberty is associated with a higher depression risk in adulthood (Copeland *et al.*, 2010), whilst the *attenuation hypothesis* predicts that differences in depression risk will attenuate due to ‘catch up’ by late developers and/or recovery by early developers (Copeland *et al.*, 2010; Senia *et al.*, 2018).

Findings of studies investigating the association between timing of puberty and depression risk in adult women are inconsistent. While some studies have identified an association between early menarche and higher depressive symptoms (Beltz, 2018; Copeland *et al.*, 2010; Graber *et al.*, 2004; Mendle *et al.*, 2018), others have not (Boden *et al.*, 2011; Gaysina *et al.*, 2015; Goering and Mrug, 2022; Herva *et al.*, 2004; Kim *et al.*, 2023; Kuo *et al.*, 2021; Natsuaki *et al.*, 2009; Opoliner *et al.*, 2014; Senia *et al.*, 2018). Inconsistencies in the findings of previous studies may be due to differences in study design: a retrospective study design (Beltz, 2018; Herva *et al.*, 2004; Kim *et al.*, 2023); self-ratings of physical development from schematic drawings (Tanner stage ratings of breast and pubic hair) (Copeland *et al.*, 2010; Goering and Mrug, 2022; Graber *et al.*, 2004; Kuo *et al.*, 2021; Natsuaki *et al.*, 2009; Senia *et al.*, 2018); inadequate confounder adjustment (Beltz, 2018; Boden *et al.*, 2011; Gaysina *et al.*, 2015; Goering and Mrug, 2022; Graber *et al.*, 2004; Kim *et al.*, 2023; Kuo *et al.*, 2021; Mendle *et al.*, 2018; Natsuaki *et al.*, 2009; Senia *et al.*, 2018); limited follow-up period (Boden *et al.*, 2011; Copeland *et al.*, 2010; Kuo *et al.*, 2021; Natsuaki *et al.*, 2009; Opoliner *et al.*, 2014) and modest sample sizes (*n* < 500) (Beltz, 2018; Boden *et al.*, 2011; Goering and Mrug, 2022).

Here we use data from a large, prospective UK birth cohort to examine whether levels of depressive symptoms from adolescence to adulthood vary according to timing of menarche. We aim to address previous study design differences with the use of a prospective birth cohort, reported age at menarche, appropriate confounder adjustment, ample sample size and adequate, repeated follow-up. Our specific aim is to test the selective persistence and attenuation hypotheses and examine whether the increased risk of depressive symptoms in early, compared with later, maturing girls persists into adulthood.

## Methods

### Participants

The sample comprised participants from the Avon Longitudinal Study of Parents and Children (ALSPAC). Pregnant women resident in the former Avon Health Authority in southwest England, having an estimated date of delivery between 1 April 1991 and 31 December 1992, were invited to take part, resulting in a cohort of 14,541 pregnancies and 13,988 children (6,762 girls) alive at 12 months of age. The phases of enrolment are outlined in Supplement 1 and described in more detail in the cohort profile paper and its update, along with details on representativeness (Boyd *et al.*, 2013; Fraser *et al.*, 2013). The total sample size for analyses using any data collected after the age of seven is 15,454 pregnancies, resulting in 15,589 foetuses. Of these, 14,901 were alive at 1 year of age. Where data were gathered from participants aged 22 and onwards, study data were collected and managed using Research Electronic Data Capture (REDCap) electronic data capture tools hosted at the University of Bristol (Harris *et al.*, 2009). REDCap is a secure, web-based software platform designed to support data capture for research studies.

Detailed information about the cohort has been collected since early pregnancy, including regular self-completion questionnaires from mothers and children. Information about ALSPAC is available at www.bristol.ac.uk/alspac/; please note that the study website contains details of all the data that are available through a fully searchable data dictionary and variable search tool (http://www.bristol.ac.uk/alspac/researchers/our-data/).

### Measures

#### Pubertal timing

A series of nine postal questionnaires pertaining to pubertal development were administered approximately annually when the study child was of age 8 to 17 years. The questionnaires asked whether menstruation had begun and if so, at what age (in years and months). Consistent with earlier studies, the first-reported age at menarche was used because these data should be the least affected by recall error, and a three-level categorical timing of menarche variable was derived: ‘early’ (before the age of 11 years and 6 months), ‘normative’ (at or after 11 years and 6 months and before 13 years and 6 months) and ‘late’ (at or after 13 years and 6 months) (Joinson *et al.*, 2013) (Table 1).

**Table 1.** Frequency of participants who experience menarche at each age in years

Age at menarche (years)	Frequency
7	1
8	13
9	42
10	243
11	883
12	1423
13	1133
14	456
15	81
16	16

#### Depressive symptoms

The Short Mood and Feelings Questionnaire (SMFQ) (Angold *et al.*, 1995) is a brief (13-item) self-report questionnaire enquiring about depressive symptoms occurrence over the past 2 weeks. It correlates highly with the Children’s Depression Inventory and the Diagnostic Interview Schedule for Children and has been shown to be a valid instrument for capturing depressive symptoms (Angold *et al.*, 1995; Turner *et al.*, 2014). This study uses SMFQ data from nine time points assessed at mean ages 12.8 to 25.8 years (hereafter referred to as 13 to 26 years) (Table S1). Further details can be found in Supplement 1.

#### Confounders

Confounders were chosen based on empirical evidence of factors that increase the risk of both early timing of menarche and depressive symptoms, and this was based on previous studies of ALSPAC and the literature of these factors in other studies. Confounders were derived mainly from maternal questionnaires completed in the first 5 years of the study child’s life. The use of ALSPAC data allowed us to include all known confounders of age at menarche and depressive symptoms, and these may be more accurately measured compared to previous papers given the prospective design and use of clinics.

They included social class, major financial problems, home ownership status and maternal educational qualifications. Models were also adjusted for absence of biological father (Culpin *et al.*, 2015, 2014) and child’s body mass index (BMI) (Cortese *et al.*, 2009; Sloboda *et al.*, 2007). Further definitions for these confounders are given in Supplement 1.

### Statistical analysis

All statistical analyses were performed in Stata (versions 15, 16) and Mplus (version 8.3).

#### Multilevel models

We used multilevel models to examine change in depressive symptoms over time with reduced measurement error, to incorporate data for participants’ SMFQ data at some but not all ages and to enable us to incorporate the age variation at each wave as previously explained (Kwong *et al.*, 2021).

This analysis estimated a pair of single-level models that used all the available SMFQ and age data to understand the population mean pattern of change in depressive symptoms. This analysis ignored the within-person clustering in the data. We used two approaches, fractional polynomials (FP) and linear splines, and these approaches produced a set of basic functions and spline terms, respectively. These were used in a pair of multilevel models. Further details can be found in Supplement 2.

FP models are advantageous as they produce a smoother model fit and have an automated method for selecting the best fitting model. However, they are limited as coefficients are harder to interpret, in comparison linear spline models are computationally simple and estimates are more easily interpretable compared to more complex FP models.

First, we fitted a FP model to understand the pattern of the longitudinal curve of depressive symptoms from early adolescence to adulthood prior to the addition of timing of menarche groups using a standard approach (Howe *et al.*, 2016) (Figure S1). We then used this FP model to build a linear spline model. Further details can be found in Supplement 2. We assessed model fit of FP and linear spline model using Akaike Information Criterion, Bayesian Information Criterion and deviance and found that the linear spline model was a better fit (Table S2).

Age was centred at 13 years, the earliest SMFQ time point. We then estimated differences in SMFQ scores between participants with an early timing of menarche compared to normative, and late timing of menarche compared to normative with and without confounder adjustment.

While we assess the patterns of depressive symptoms over time, we specifically focus on ages 14, 18 and 24 years because these ages correspond to (1) mid-adolescence (when there are high levels of variability in pubertal maturation between girls), (2) late adolescence (when most girls have experienced menarche) and (3) young adulthood (when all girls are post menarche). While we used information at age 26 to model the patterns of depressive symptoms using multilevel linear spline models, the precise estimates for age 26 were not chosen to demonstrate the long-lasting effects of timing of menarche in young adulthood, instead we focused on age 24. This is because the different nonlinear models did not adequately fit the data and yielded the greatest differences at the extremes of the measured age range.

### Imputation

#### Imputation for linear spline models

Whilst the longitudinal models described above can address the problem of incomplete depressive symptom data through maximum likelihood methods, this fails to account for incomplete exposure data. To address this problem, we used Mplus and Markov chain Monte Carlo estimation. First, the linear spline model was re-estimated in Mplus using Bayesian estimation, then auxiliary data were incorporated and allowed to co-vary with the exposures from the substantive model. Further details are described in Supplement 2. We focused on the imputed linear spline model to avoid bias and reduced power arising from incomplete exposure data.

### Sensitivity analyses

While multilevel models provide more detail on the patterns of depressive symptoms over time, examining simplistic models may put confidence in the findings from complex models. Therefore, we examined the association between timing of menarche and depressive symptoms at ages 14, 18 and 24 years (separately) in linear regression models with and without adjustment for confounders. We imputed the missing SMFQ data and confounders to minimise selection bias. Further details can be found in Supplement 1.

## Results

Table 2 shows the distribution of confounders across early, normative and late timing of menarche groups for participants who provided SMFQ data from at least one time point. Amongst girls with early menarche compared with those with normative and late menarche, there was a higher proportion of father absence, living in rented accommodation and higher BMI and a lower proportion of girls with low BMI. Compared with the normative group, early and late menarche was associated with a higher proportion of mothers with low educational attainment.

**Table 2.** Distribution of confounders across timing of menarche groups

Confounders	Early menarche, *N* (%) = 622 (15.30)	Normative menarche, *N* (%) = 2453 (60.34)	Late menarche, *N* (%) = 990 (24.35)	*P* value (chi^2^)
Father absence (% under age 5 (*n*))	24.0 (117)	15.1 (298)	14.1 (114)	<0.001
Father absence (% age 5 or older (*n*))	9.6 (47)	8.3 (163)	7.8 (63)	0.498
Home ownership (% rented from council or housing association or being bought from council (*n*))	15.3 (75)	9.9 (200)	12.2 (102)	0.002
Major financial problems (% yes (*n*))	28.4 (159)	26.8 (605)	25.5 (234)	0.475
Social class (% lower skilled occupations (*n*))	6.1 (32)	4.7 (101)	4.3 (37)	0.287
Maternal education (% mother with GCSEs below C grade, vocational qualification, undertaken an apprenticeship, is a state enrolled nurse, has city and guilds qualifications or has no qualifications (*n*))	26.2 (147)	22.7 (510)	31.1 (282)	0.024
Lower child BMI, (% yes (*n*))	6.6 (33)	11.7 (237)	25.3 (210)	<0.001
Higher child BMI (% yes (*n*))	39.5 (197)	20.6 (417)	9.3 (77)	<0.001

**Figure 1.**
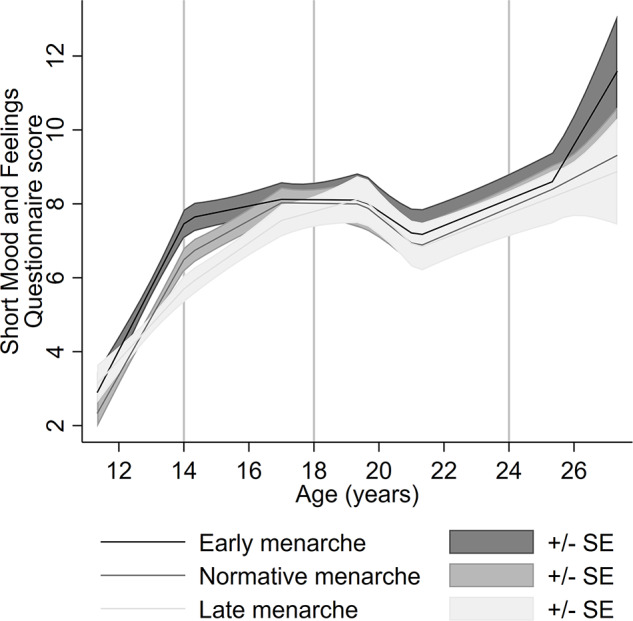
Adjusted pattern of depressive symptoms from ages 13 to 26 across three timing of menarche groups using imputed linear splines.

Table S3 shows the mean SMFQ scores and distribution of timing of menarche and confounders across different levels of missing data on the SMFQ. Completion of the SMFQ at fewer time points was associated with higher depressive symptom scores at age 13, 18, 23 and 24 years; early timing of menarche; father absence; living in rented accommodation; lower social class; lower maternal education and higher BMI. Figure 1 shows the imputed confounder-adjusted level of depressive symptoms across the three timing of menarche groups. At age 14, the level of depressive symptoms is higher for the early timing of menarche group compared to normative, while the level of depressive symptoms is lower for the late timing group. By age 18, depressive symptom levels for the normative and late timing groups caught up with the early menarche group. After which, for all timing of menarche groups, depressive symptoms decreased until the early twenties and then increased again. At age 26, levels of depressive symptoms continue to increase across all menarche groups; however, this increase is larger for the early menarche group, although evidence is not conclusive due to overlapping confidence intervals. This lack of certainty may be due to the sparsity of data at age 26 and because longitudinal models perform poorly in the tails of the age distribution.

**Figure 2.**
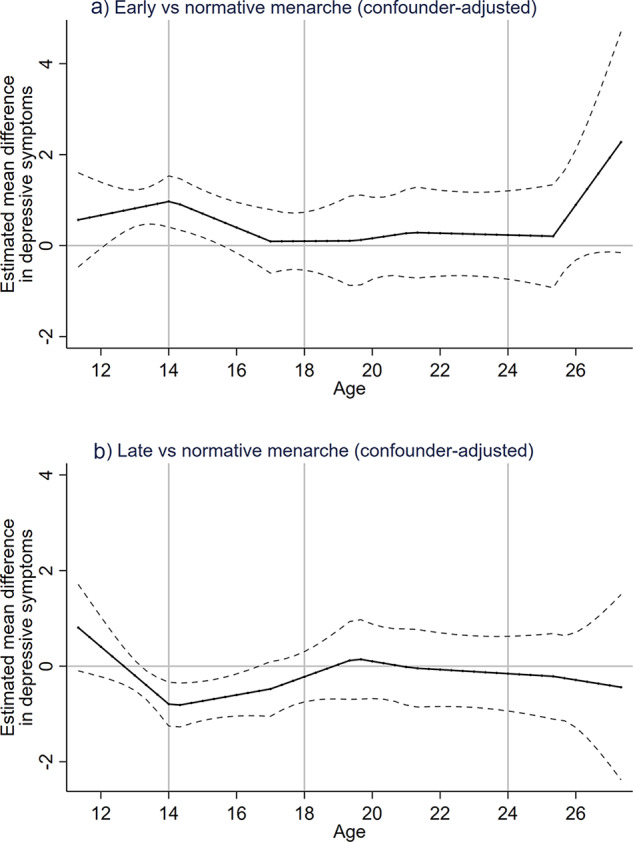
Estimated mean difference in depressive symptoms between timing of menarche groups using adjusted imputed linear splines. Dashed lines indicate 95% confidence intervals. Panels show a) estimated mean difference in depressive symptoms of early compared to normative menarche and b) estimated mean difference in depressive symptoms of late compared to normative menarche.

The estimated differences between timing groups for the imputed confounder-adjusted model demonstrate this further, indicating depressive symptoms are ∼1 point higher in the early timing of menarche group compared to normative and ∼0.7 point lower in the late timing group, compared with the normative timing group in mid-adolescence (Table 3, Fig. 2). In young adulthood, we did not find evidence to suggest a difference between the late and normative timing of menarche groups, whereas there appears to be a sudden increasing difference between the early timing group compared to normative timing of menarche at age 26 (Fig. 2a). However, as noted above, confidence intervals span the null. Unadjusted imputed findings are consistent with the adjusted imputed findings, except at age 26 where there is evidence for a positive difference between early and normative menarche in the unadjusted model but not in the adjusted model (Table S4, Figure S2–3).

**Table 3.** Imputed linear spline model estimated differences for both the unadjusted and adjusted models at ages 14, 18 and 24 years (*N* = 4065)

*Model*	Age (y)	Early vs. normative (95% CI)	Late vs. normative (95% CI)	Probs > chi^2^
*Unadjusted*	14	1.08 (0.58, 1.59)	−0.74 (−1.15, −0.33)	<0.0001
	18	0.37 (−0.25, 0.98)	−0.39 (−0.89, 0.12)	0.09
	24	0.46 (−0.49, 1.40)	−0.22 (−0.98, 0.54)	0.45
*Adjusted*	14	0.94 (0.44, 1.45)	−0.69 (−1.10, −0.29)	<0.0001
	18	0.10 (−0.53, 0.72)	−0.27 (−0.79, 0.24)	0.51
	24	0.24 (−0.72, 1.19)	−0.15 (−0.92, 0.62)	0.79

The findings from the linear regression analysis and those from the non-imputed linear spline model were consistent with the findings from the imputed linear spline model (Table S4; Figure S4).

## Discussion

Consistent with earlier studies, we found strong evidence that girls with an earlier timing of menarche had higher levels of depressive symptoms in mid-adolescence than those with normative and late timing (Alcala-Herrera and Marvan, 2014; Galvao *et al.*, 2014; Mendle *et al.*, 2019; Skoog *et al.*, 2016). At age 18, however, there was no evidence for differences in depressive symptom levels whereby the normative and late onset groups appeared to “catch up” with the early onset group, consistent with the *attenuation* hypothesis (Senia *et al.*, 2018). By age 20, depressive symptom levels decreased across all groups, subsequently followed by an increase, which continued from age 25 to 26 for all groups. Although the largest increase in depressive symptoms at the last time point was observed for the early timing group, there was limited evidence of a difference in depressive symptom levels according to timing of menarche, with overlapping confidence intervals. In addition, precision of estimates at this final time point was suboptimal; this was likely due to the sparsity of data at this age range. Future studies should examine whether differences in depressive symptoms levels according to timing of menarche emerge further into adulthood.

Strengths of this study include the prospective design and availability of repeated data on age at menarche and depressive symptoms in a large contemporary cohort. This allowed us to examine the long-term effects of early timing of menarche on depressive symptoms from adolescence to adulthood.

The study also has limitations that should be considered when interpreting the findings. These include sample attrition, missing data and incomplete data on confounders. The current study comprised a lower proportion of participants with socioeconomic disadvantage status compared to those who were lost to follow-up. Socioeconomic disadvantage is associated with an earlier timing of menarche and increased risk of depressive symptoms (Freeman *et al.*, 2016); therefore, our sample will underrepresent these individuals, leading to potentially biased results (Mendle *et al.*, 2016). To address potential bias, we focus on the imputed findings and found that the results from the imputed data were similar to the complete case analysis. The ALSPAC cohort is predominantly white and affluent (Boyd *et al.*, 2013; Fraser *et al.*, 2013); hence, we are unable to generalize our results to minority ethnic groups and less affluent populations. Further research in these underserved populations is vital to prevent widening inequalities in health research. Research in non-UK samples is needed to examine if these findings generalize to young people from other countries.

Our study did not find strong evidence for an association between early timing of menarche and depressive symptoms in adulthood, which was consistent with a recent umbrella review (Lee *et al.*, 2022) and prospective (Boden *et al.*, 2011; Gaysina *et al.*, 2015; Goering and Mrug, 2022; Kuo *et al.*, 2021; Natsuaki *et al.*, 2009; Opoliner *et al.*, 2014; Senia *et al.*, 2018), and retrospective studies (Herva *et al.*, 2004; Kim *et al.*, 2023). However, this finding was inconsistent with other prospective studies (Copeland *et al.*, 2010; Graber *et al.*, 2004; Mendle *et al.*, 2018) and a retrospective study, which report evidence of a persisting effect into adulthood (Beltz, 2018). In addition, we did not find evidence for an association between late timing of menarche and depressive symptoms in adulthood, which is inconsistent with prospective studies (Gaysina *et al.*, 2015; Goering and Mrug, 2022) and retrospective studies (Beltz, 2018; Herva *et al.*, 2004).

The inconsistencies with previous studies may be due to limitations in the current study, including the sparse data at the last time point or bias due to loss to follow-up. However, it may be due to differences in the methods of previous studies that might explain why our results were inconsistent; these include the use of physical development from schematic drawings as a measure of timing of puberty (Copeland *et al.*, 2010; Goering and Mrug, 2022; Graber *et al.*, 2004), lack of adjustment for confounders: childhood adiposity; social status or father absence (Beltz, 2018; Gaysina *et al.*, 2015; Goering and Mrug, 2022; Graber *et al.*, 2004; Mendle *et al.*, 2018), and modest sample sizes (Beltz, 2018; Goering and Mrug, 2022).

It is notable that the current study observed a decline in levels of depressive symptoms between ages 18 and 22, which has been shown in ALSPAC by a previous study (Kwong *et al.*, 2019). This pattern is not specific to ALSPAC; a decline in depressive symptoms in the early to mid-twenties has been identified in other cohorts and in both sexes (Adkins *et al.*, 2009; Ferro *et al.*, 2015; Ge *et al.*, 2006; Natsuaki *et al.*, 2009; Rawana and Morgan, 2014). A decline in levels of depressive symptoms from adolescence to early adulthood may be due to increased independence, perceived sense of control or establishment of stable relationships (Adkins *et al.*, 2009; Ge *et al.*, 2006). The subsequent increase in depressive symptoms in the ALSPAC cohort at age 26 has not previously been observed in other cohorts; therefore, further studies are needed to confirm this. Although the current study did not find strong evidence for a relationship between early or late timing of menarche and depressive symptoms in women in their mid-twenties, it is possible that differences in the risk of depressive symptoms in adulthood emerge after this age, as risk factors for depression change over the lifecourse (Kessler, 2003).

The findings in this study are consistent with onset of menarche being a period of increased risk of depressive symptoms, and this increase occurs irrespective of the timing of onset. Fluctuations in sex hormones including oestrogen have been linked to changes in mood and increases in depressive symptoms during puberty (Angold *et al.*, 1999; Hoyt and Falconi, 2015). Post-puberty, oestrogen levels stabilize into the typical menstrual cycle and therefore may have less influence on depressive symptoms.

Dysregulation of the hypothalamic–pituitary–adrenal (HPA) axis (involved in the body’s biological stress response) might also explain the rising levels of depression during puberty. At the onset of puberty, the HPA axis matures and its activity increases, becoming more sensitive. The combination of this increased sensitivity and increased exposure to stressors during puberty can lead to HPA axis dysregulation, mood instability and increased risk of depression (Roberts and Lopez-Duran, 2019; Steiner *et al.*, 2003).

Psychosocial theories propose that the social consequences of puberty explain more of the variance in adolescent depression than biological changes (Brooks-Gunn and Warren, 1989). It may be that physical development of a girl’s body from the onset of puberty increases the risk of unwanted sexual attention from peers, which in turn, could lead to feelings of body shame (Lindberg *et al.*, 2007; Petersen and Hyde, 2009). One study has found that a more advanced breast development stage at age 14 is associated with increased depressive symptoms later in adolescence irrespective of the timing of puberty (Lewis *et al.*, 2018). The ‘catch up’, in terms of depressive symptoms, by the normative and late timing groups by the end of adolescence may be due to improved psychological well-being and maturity for all girls as they enter adulthood (Caspi *et al.*, 2005; Schulenberg *et al.*, 2005).

## Conclusions

We did not find evidence to support the *selective persistence hypothesis*, but instead we found that girls with later timing of menarche catch up with early maturing girls in depressive symptom levels. Hence, the observed differences in adolescent depressive symptom levels between girls with early, normative and late timing of puberty attenuate in young adulthood. Our results imply that the onset of menarche is a period of increased risk of depressive symptoms irrespective of the timing of onset, and therefore, it is important to provide a supportive environment for all girls at this developmental transition. There is evidence that inadequate and poorly timed puberty education can lead to psychological distress (Herbert *et al.*, 2017), and concerns have been raised (e.g., by the Personal, Social and Health Education association) that new English secondary school curricula on ‘*Relationships and Sex Education, and Health Education*’ (mandatory from September 2020) lack detail on puberty and are delivered too late. Evidence-based psychoeducational materials as well as practical and emotional mental health support are needed to prepare young people for the pubertal transition during this sensitive period.

## Supporting information

Prince et al. supplementary material 1 Prince et al. supplementary material

Prince et al. supplementary material 2 Prince et al. supplementary material

Prince et al. supplementary material 3 Prince et al. supplementary material

## Data Availability

Data from the Avon Study of Parents and Children is available to researchers through an online proposal system. Information regarding access can be found on the ALSPAC website (http://www.bristol.ac.uk/media-library/sites/alspac/documents/researchers/data-access/ALSPAC_Access_Policy.pdf).

## Supplementary material

The supplementary material for this article can be found at https://doi.org/10.1017/S2045796023000707.
