# Supplementary material for: The relationship between timing of onset of menarche and depressive symptoms from adolescence to adulthood
Source: Epidemiol Psychiatr Sci. 2023 Sep 28;32:e60. doi: 10.1017/S2045796023000707 (PMC10539742; doi:10.1017/S2045796023000707)
Supplement: Prince et al. supplementary material 1 — Prince et al. supplementary material [file S2045796023000707sup001.docx]

# Supplement 1

## Participants – phases of enrolment

When the oldest children were approximately 7 years of age, an attempt was made to bolster the initial sample with eligible cases who had failed to join the study originally. As a result, when considering variables collected from the age of seven onwards (and potentially abstracted from obstetric notes) there are data available for more than the 14,541 pregnancies mentioned above. The number of new pregnancies not in the initial sample (known as Phase I enrolment) that are currently represented on the built files and reflecting enrolment status at the age of 24 is 913 (456, 262 and 195 recruited during Phases II, III and IV respectively), resulting in an additional 913 children being enrolled (Northstone *et al.*, 2019).

## Measures

### Depressive symptoms

This study uses SMFQ data from 9 timepoints assessed at ages 12.8 to 25.8 years (hereafter referred to as 13 to 26 years). **Table S1** shows considerable variability around the mean age at completion, however it has previously been reported that variability in age at completion of the SMFQ at each time point in the ALSPAC cohort is not related to level of depressive symptoms (Joinson *et al.*, 2013).

### Confounders

Social class was defined as the lower of the mother’s or partner’s occupational social class and dichotomised into classes I-IV, professional, managerial, or skilled professions, and V-VI, partly skilled or unskilled professions

Homeownership status was defined as living in owned or privately rented accommodation versus subsidised housing

Maternal education qualifications were defined as none versus high school qualifications or greater), and major financial problems (yes/no).

Biological father absence was categorised by father being present, father not living with the child when the child was less than 5 years old, and father not living with the child when the child was more than 5 years old, and less than 10 years old. Father absence was categorised in this way as there is evidence that father absence before the age of 5 is more strongly associated with depressive symptoms compared to later in childhood (Culpin *et al.*, 2014; Alvergne *et al.*, 2008).

Child’s BMI was obtained from height and weight measurements obtained during a research clinic attended at age 9 years (three-level categorical BMI variable: lowest BMI <15; mid-range BMI 15–20; highest BMI >20).

## Linear regression analyses

We perform linear regression analysis of age at menarche on depressive symptoms at ages 14, 18 and 24. The analysis included participants who reported their age at menarche and complete SMFQ information.

## Imputation for linear regression models

Imputation for the individual linear regression models was performed in Stata using Full Conditional Specification (ice reference) (StataCorp LP., 2011). This was performed on the 4065 individuals who reported their age at menarche and completed the SMFQ questionnaire for at least one timepoint.

Missing data for the nine SMFQ measures was imputed using prediction mean matching whilst confounders were imputed using either binary logistic, multinomial logistic or ordinal as appropriate. A series of additional auxiliary variables, comprising parity, family income, maternal substance use at age 12, and young person’s substance use at age 13 were included. Twenty cycles of regression switching were used, and 100 imputed datasets were produced. Monte Carlo errors were examined as a guide for determining an appropriate number of imputed datasets.

## References

**Alvergne A, Faurie C and Raymond M**. (2008) Developmental plasticity of human reproductive development: effects of early family environment in modern-day France. *Physiol Behav* **95**: 625-632.

**Culpin I, Heron J, Araya R, Melotti R, Lewis G and Joinson C**. (2014) Father absence and timing of menarche in adolescent girls from a UK cohort: the mediating role of maternal depression and major financial problems. *J Adolesc* **37**: 291-301.

**Joinson C, Heron J, Araya R and Lewis G**. (2013) Early menarche and depressive symptoms from adolescence to young adulthood in a UK cohort. *J Am Acad Child Adolesc Psychiatry* **52**: 591-598 e592.

**StataCorp LP.** (2011) *Stata multiple-imputation reference manual : release 12,* College Station, Tex.: Stata Press.
