# Supplementary material for: The relationship between timing of onset of menarche and depressive symptoms from adolescence to adulthood
Source: Epidemiol Psychiatr Sci. 2023 Sep 28;32:e60. doi: 10.1017/S2045796023000707 (PMC10539742; doi:10.1017/S2045796023000707)

# Supplementary figures


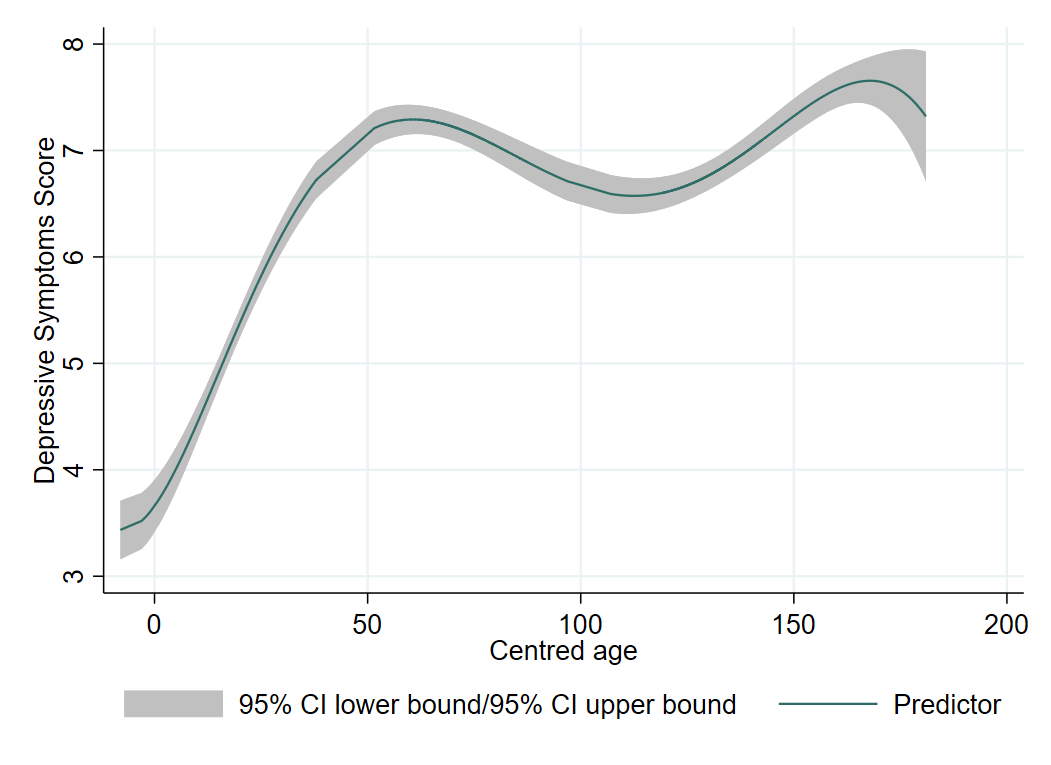


Knot point

**Figure S1** Fractional polynomial growth curve. O indicates knot points selected for the linear spline model. Age is centred at 12.8 years – the earliest SMFQ timepoint and is shown here in months.


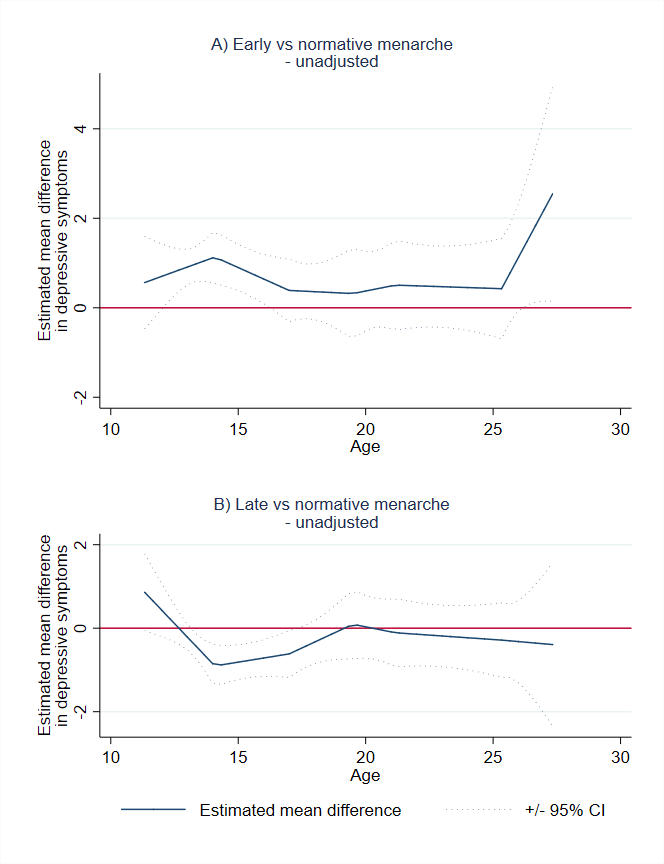


**Figure S2** Estimated mean difference in depressive symptoms between timing of menarche groups using unadjusted imputed linear splines. X axis shows age in years. Panels show **A)** estimated mean difference in depressive symptoms of early compared to normative menarche and **B)** estimated mean difference in depressive symptoms of late compared to normative menarche.


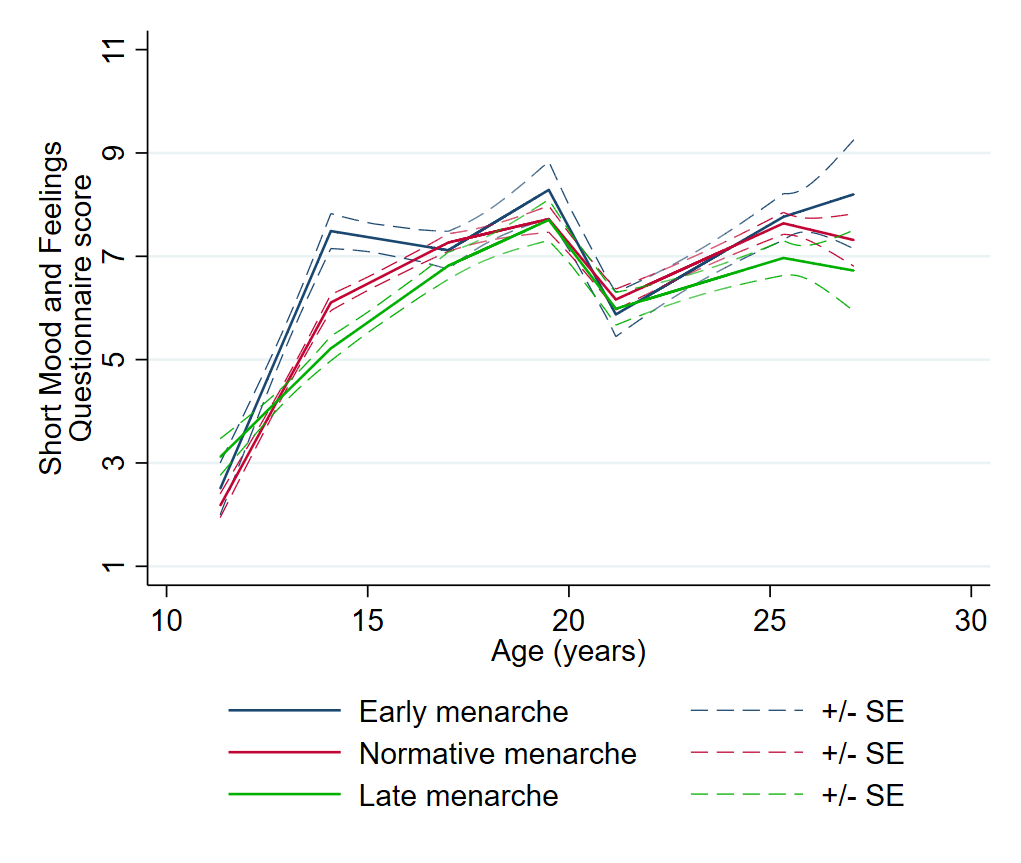


**Figure S3** Unadjusted pattern of depressive symptoms from ages 13 to 26 across three timing of menarche groups using imputed linear splines. X axis shows age in years.

**Figure S4** Estimated mean differences in depressive symptoms between timing of menarche groups using non-imputed linear splines. X axis shows age in years. Panels show **A)** unadjusted and **B)** adjusted estimated differences of **early** compared to normative menarche and **C)** unadjusted and **D)** adjusted estimated differences of **late** compared to normative menarche.


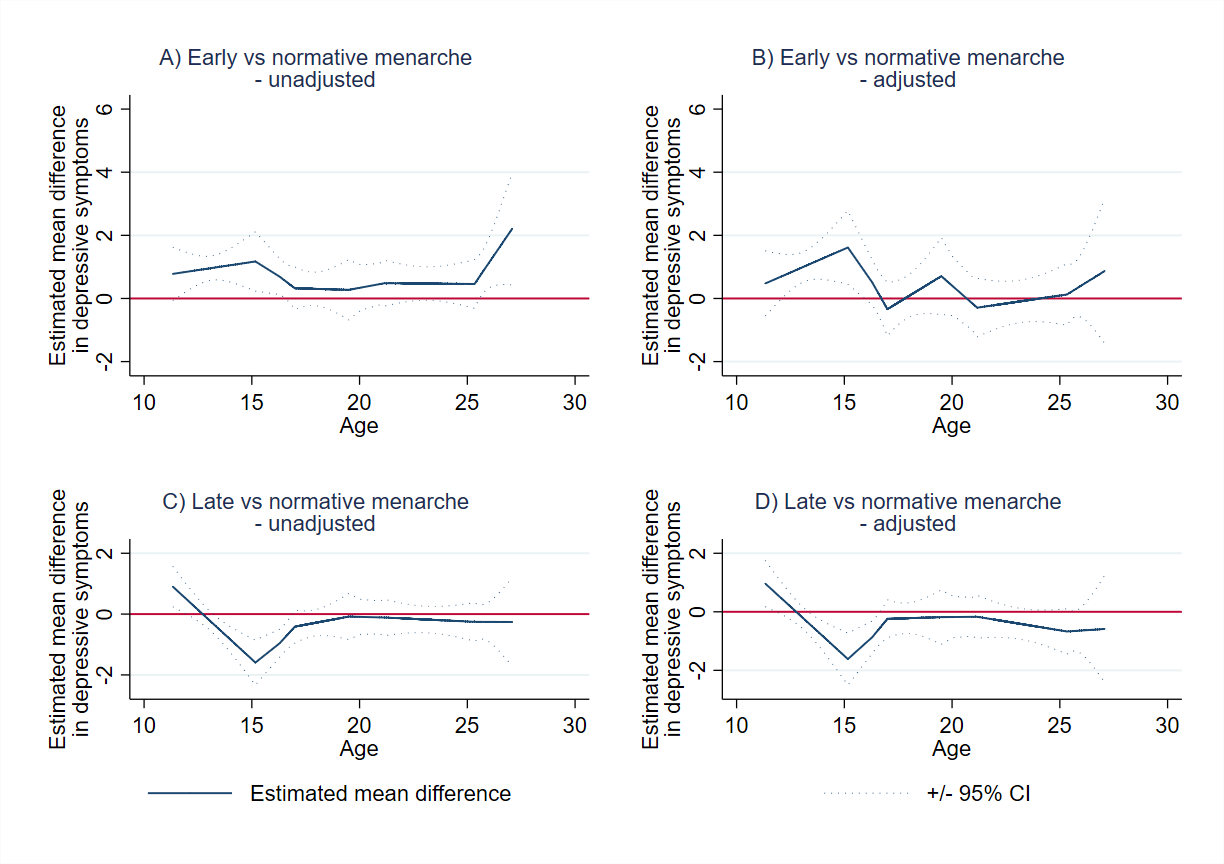

Supplement: Prince et al. supplementary material 2 — Prince et al. supplementary material [file S2045796023000707sup002.docx]
